# Fitness-to-work considerations in the paradigmatic pain condition of headache disorder

**DOI:** 10.1186/s10194-025-01960-1

**Published:** 2025-01-31

**Authors:** Xiangning Fan, Ellina Lytvyak, Charl Els, Sebastian Straube

**Affiliations:** 1 Department of Medicine, Faculty of Medicine and Dentistry, University of Alberta, Edmonton, AB Canada; 2 Department of Psychiatry, Faculty of Medicine and Dentistry, University of Alberta, Edmonton, AB Canada; 3 School of Public Health, University of Alberta, Edmonton, AB Canada

**Keywords:** Fitness-to-work, Headache disorders, Occupational health

## Abstract

Headache disorders are common, including in the working population. Clinicians caring for patients with headache need to be aware of work-related factors as potential causes or triggers of headache disorders, and consider the impact of headache on fitness-to-work, especially in safety-sensitive and decision-critical roles. Such fitness-to-work determination should include individualized consideration of the nature of the headache disorder itself, the pattern of the headache, the impact of sleep deprivation on the headache as it relates to fitness to do shiftwork, medication and substance side effects, fitness-to-work implications of associated medical or psychiatric conditions, and the potential of symptom feigning or malingering for secondary gain. As clinicians often struggle with fitness-to-work determinations, a structured approach to fitness-to-work assessments in headache conditions and other pain conditions would improve clarity for clinicians and increase the quality of care provided to patients, with potential benefits for workplace safety and policy in this arena as well.

## Background and significance

Headache disorders affect approximately 40–50% of the global population, impacting health, quality of life and occupational performance of people across their lifespan [1–3]. Their prevalence has increased substantially over the last three decades, with almost 40% of the rise observed in a younger population [4]. Across all age categories and both genders, headache disorders currently account for 1.65% of total disability-adjusted life-years (DALYs) [3]. Years lived with disability (YLDs) attributed to all headache disorders increased by over 14% between 2010 and 2021 and are now ranked third among the largest contributors to all-cause YLDs [3]. While numerous types of primary and secondary headache are commonly encountered, the two dominant primary disorders are tension-type headache (TTH) and migraine [5]. While TTH is more common, migraine accounts for the majority of headache-related disability and is the leading cause of days lost due to disability worldwide among individuals under the age of 50 [1].

Headache disorders represent a major, but often overlooked, cause of lost work time and productivity as well as disability, under- and unemployment [6, 7]. Both migraine and TTH are more prevalent among people in their most productive years, peaking in prevalence between ages 25 and 55 [8].

Headache disorders may also be attributed to some occupational activities and exposures. Evidence suggests an association between headache and shift work, use of computer monitors, inadequate lighting, poor indoor air quality, and odours – which may all be encountered at the workplace – as well as long working hours [9–14]. Workplace exposure to chemicals (lead, micro-pollutants, and tobacco smoke), physical factors (noise, vibration), and select personal protective equipment, such as face masks, may also cause headaches or trigger attacks in those with pre-existing migraine or TTH [15–20]. Headache disorders may also arise from workplace injuries, such as head trauma or neck injury [21]. Psychological factors, such as work-related stress, increased demand, insufficient control over work, bullying, injustice, effort-reward imbalance, and exposure to certain leadership styles may contribute to, or exacerbate a worker’s headaches, adversely impacting productivity, and resulting in absenteeism and presenteeism [22–25].

Regardless of the association between occupational factors and headache disorders, the workplace and fitness-to-work are inconsistently considered in the clinical assessment of individuals with headache disorders [26], and both general and specialist physicians are often not specifically trained in or comfortable at considering workplace factors in medical assessments [27, 28]. As far as we are aware, there is no published framework for fitness-to-work evaluations in this patient population. Given the common occurrence of headache disorders in the working population, it is likely that many individuals in safety-sensitive and decision-critical [29] jobs will experience headache disorders. While it is not possible to offer an exact estimate of the number of workers engaged in safety-sensitive and decision-critical work (please see the glossary included with this paper for definitions) globally, a fitness-to-work approach [30] in the evaluation of the individual patient with a headache disorder is important to protect workplace safety while enabling the worker to participate as fully in the workforce as possible.

Notwithstanding the existence of various approaches to fitness-to-work [31], a commonly used framework is that of the American Medical Association [32], which categorizes fitness-to-work issues into those resulting in increased risk of harm at the workplace, those resulting in decreased worker capacity to do work when compared to essential job demands, and those impacting workers’ tolerance of the work; these concepts are described in the glossary included with this paper. While headache disorders may impact any of these three factors, fitness-to-work assessments can only assess issues of risk and capacity [32]. Work restrictions and limitations should be assessed individually, and based on the impact of the condition and co-occurring conditions on occupational capacity and risk. Occupational tolerance may also be adversely impacted. Unlike the case with headache impacting occupational risk and capacity, headache impacting occupational tolerance alone may not trigger the employer’s duty to accommodate in all cases. Due to recognition as a disability under various compensation and legislative schemes, headache disorders may trigger legislative protections for disability assessment and workplace accommodations in many jurisdictions. Adjudication of disability related to headache disorders is complicated, given that pain is a subjective phenomenon.

**Fig. 1:**
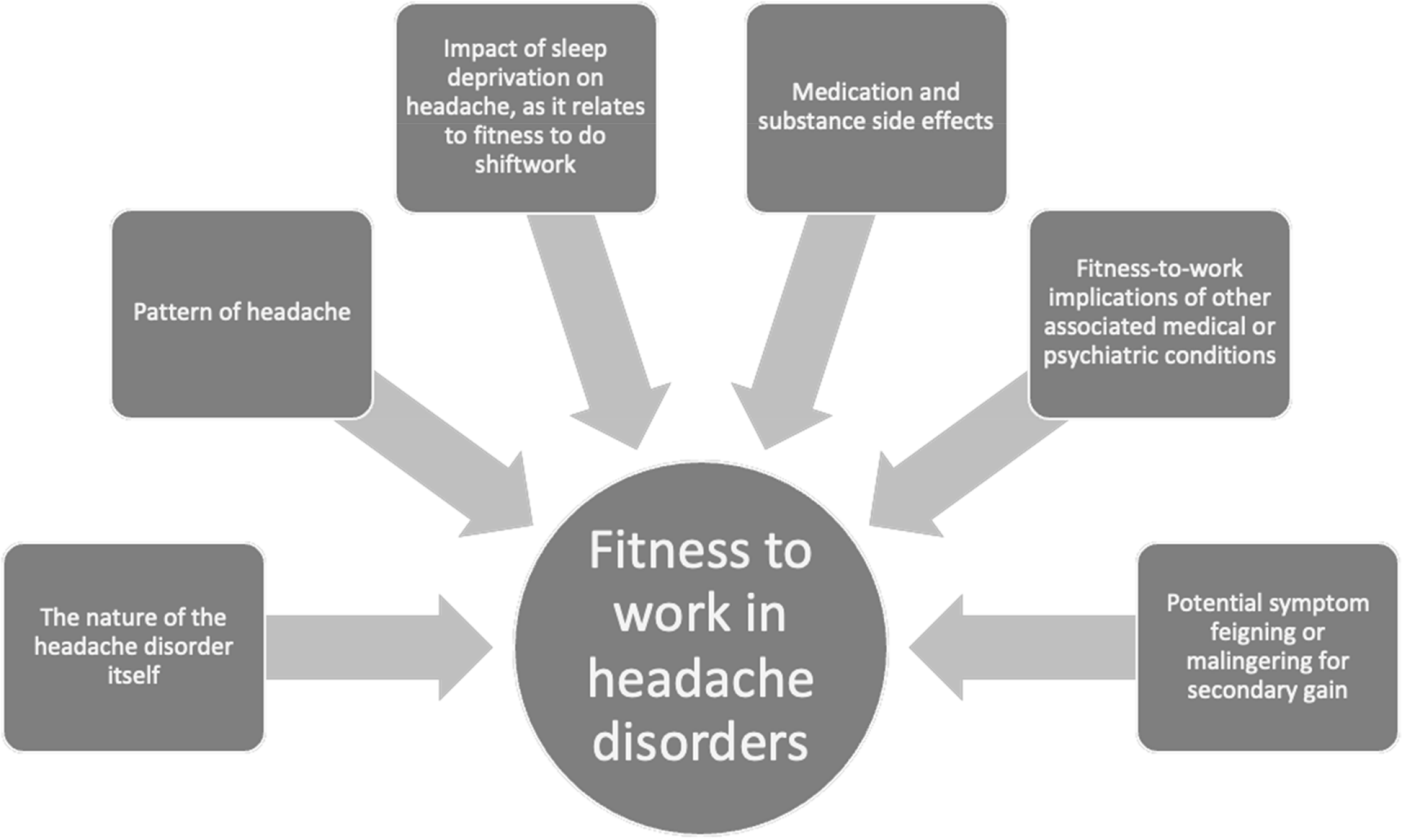
Fitness-to-work considerations in headache disorder

Fitness-to-work considerations with respect to headache disorders can be broadly grouped into the categories illustrated in Fig. 1, which are discussed further in the remainder of this paper.

Failure to achieve maximum medical improvement should trigger an investigation of the diagnosis and its cause(s) or origin, the presence of co-occurring conditions, as well as an assessment for a surreptitious substance use disorder, or malingering, and the degree of adherence to evidence-based care and treatment.

### The nature of the headache disorder itself

The severity of headache pain and associated manifestations, particularly if there are neurological symptoms that are function-limiting, may impact work ability. With migraine, associated symptoms such as visual disturbance, nausea/vomiting, motor weakness, confusion or speech disturbance may be as or more function-limiting than the headache itself. Perhaps for this reason, as well as the severity of pain symptoms that is a defining feature of migraine, migraine is reported to account for the vast majority of headache-related disability when compared to other types of headache [33].

If there is an aura, the nature and duration of the aura are important in fitness-to-work determinations. Impairing symptoms that arise quickly at the start of a headache episode are of more concern from a fitness-to-work perspective than an aura that allows a worker some time during which they remain functional and able to wrap up work tasks before being incapacitated. Therefore, a key determinant of risk – to be considered in the context of the workplace in question – is the duration of the prodromal symptoms that are not in and of themselves impairing but that indicate upcoming impairment.

### Pattern of headache

While headache is typically transient, it can be recurrent. The extent to which a headache may interfere with work depends on the type, frequency and intensity of the headache, on the individual with their comorbidities, and on the nature of the work. Some individuals may be able to manage quite well in the workplace despite symptoms, while others with more severe pain or more frequent headaches may struggle, causing workplace performance to suffer and, in safety-sensitive and decision-critical roles, presenting a potential safety or other risk at work.

Individuals with poorly controlled headache disorders may also present with frequent and unpredictable work absences, leading to workplace attempts to manage absences through various disciplinary and non-disciplinary mechanisms, if not overt job termination where workplace protections for employees are not strong. Alternatively, they may be present at the workplace, but with reduced workplace productivity. Presenteeism, for example, rather than absenteeism is the predominant issue with migraine [7, 33, 34].

### Impact of sleep deprivation on headache, as it relates to fitness to do shiftwork

Sleep disorders are associated with a number of headache disorders [35], and sleep deprivation precipitates or worsens headaches through a number of potential mechanisms [36, 37]. A relationship between shift work, night work and headache has been described in the literature [38, 39], although the data are conflicting, and, in this context, the patient may require careful evaluation if their work schedule comprises working rotating or night shifts, both in relation to their fitness to perform shiftwork as well as the impact of shiftwork on the headache disorder.

### Medication and substance side effects

The impact of both abortive and suppressive medications for headaches may have workplace implications. Medications that are sedating or which affect cognitive function are perhaps of the most concern. Complicating this matter, the use of pain medications, particularly those available over the counter or used for self-medication, may not be readily volunteered by workers. Among pain-relieving medications, opioids are particularly noteworthy from a fitness-to-work perspective due to their side effect profile and increased risk for a substance use disorder [40], and the use of opioids for the treatment of chronic non-cancer pain is generally not supported. Some national professional societies in occupational medicine consider opioid medication use to be not recommended for safety-sensitive work [41]. Complications related to other pain medications (for example, gastrointestinal bleeds related to the use of non-steroidal anti-inflammatory medications) may also impact fitness-to-work.

Cannabis products – whether licit or illicit, whether authorized for medical purposes, or obtained as recreational cannabis and then used by a worker to also self-medicate – are often utilized with the aim of pain relief, including for headache, and they may impact the ability to perform safety-sensitive work [42]. As with opioids, some national professional societies in occupational medicine explicitly advise against the use of cannabis for persons in safety-sensitive positions without an appropriate washout period [43]. Comorbid use of other substances (e.g. alcohol or other recreational drugs) may additionally have fitness-to-work implications, or trigger headaches in their own right [44].

### Fitness-to-work implications of other associated medical or psychiatric conditions

Headache disorders may not occur in isolation. Traumatic head injury, for example, is a frequent cause of secondary headaches that may have important fitness-to-work implications in its own right with respect to cognitive capacity, judgement and decision-making ability and worker insight into the condition and their own limitations. Headache disorders commonly co-occur with psychiatric conditions (e.g. mood disorders, anxiety disorders), substance-related disorders (e.g. alcohol, nicotine, and caffeine use), neurological, and cardiovascular conditions, among others, which independently may impact fitness-to-work and which warrant additional assessment.

### Symptom feigning and malingering for secondary gain

From a fitness-to-work perspective, malingering interferes with the validity of the clinical assessment. In disorders characterized primarily by patients’ subjective and self-reported experience (e.g. psychiatric disorders and many pain disorders, including headache), and where the possibility of secondary gain exists, clinicians should be alert to this possibility.

As objective physical or investigational findings may not be present in many headache disorders, diagnosis and treatment of headache may be based largely or exclusively on worker self-report. Headache disorders therefore represent a unique challenge in certain contexts (e.g. workers’ compensation or civil litigation), where elevated rates of negative distortion [45] and malingering [46, 47] have been described in the literature. Although malingering is by no means a universally observed phenomenon [48], treating healthcare providers often do not consider malingering as a cause of symptoms, even in contexts where significant secondary gain may be possible [49].

#### Conclusions

Headache may serve as a paradigmatic pain condition, which is common, may be work-related, and should be adequately considered in fitness-to-work assessments. All fitness-to-work cases are fact-specific and require individual assessment. We hope that the approach presented here will be helpful in performing such an assessment. Many of the above-mentioned considerations and the general framework will be applicable to other types of pain as well. Stakeholders need to be mindful that there may be impaired fitness-to-work due to headache and that time away from work may be needed to allow for recovery from headache and associated conditions.

## Data Availability

No datasets were generated or analysed during the current study.

## Glossary

### *Concepts from the American medical association guides to the evaluation of work ability and return to work*

Risk: “*Risk *refers to the chance of harm to the patient, co-workers, or the general public, if the patient engages in specific work activities.” [50]
Capacity: “*Capacity* refers to concepts such as strength, flexibility, and endurance. […] Actually, ‘capacity’ indicates that the individual is already maximally trained and fully acclimatized to the job or activity in question.” [50]
Tolerance: “*Tolerance* is a psychophysiologic concept. It is the ability to tolerate sustained work or activity at a given level.” [50]

### *Safety-sensitive and decision-critical work*

Safety-sensitive work: “’[S]afety-sensitive’ work can be thought of as encompassing work in which one or more ‘safety-critical’ tasks are or may be performed, and where possible consequences include death or serious injury of a worker or a member of the general public, or, alternately, damage to or serious disruption of equipment, production or the environment.” [29]
Decision-critical work: Decision-critical workers are those “whose continued occupational performance depends on the ability to consistently exercise judgment and insight, but who do not precisely fall under the ‘safety sensitive’ category. Examples of such ‘decision-critical’ workers include corporate executives, schoolteachers, lawyers, information technology workers, and some health professionals, among others.” [29]
